# Smoking cessation for peripheral arterial disease: systematic review protocol

**DOI:** 10.1590/0100-6991e-20233482-en

**Published:** 2023-03-24

**Authors:** DANIELLE AKEMI BERGARA KURAMOTO, LUIZ FERNANDO SANTETTI ZANIN, RONALD LUIZ GOMES FLUMIGNAN, CAROLINA DUTRA QUEIROZ FLUMIGNAN, MARCELLO ERICH REICHER, REBECA MANGABEIRA CORREIA, LUÍS CARLOS UTA NAKANO

**Affiliations:** 1 - Universidade Federal de São Paulo, Disciplina de Cirurgia Vascular, Departamento de Cirurgia - São Paulo - SP - Brasil

**Keywords:** Peripheral Arterial Disease, Smoking Cessation, Tobacco Use Cessation, Tobacco Use Disorder, Doença Arterial Periférica, Abandono do Hábito de Fumar, Abandono do Uso de Tabaco, Transtorno por Uso de Tabaco

## Abstract

**Background:**

peripheral arterial disease has smoking as its main avoidable vascular risk factor. However, most studies do not focus on smoking as the main exposure variable.

**Objectives:**

to assess the impact of smoking cessation interventions versus active comparator, placebo or no intervention, on peripheral arterial disease outcomes.

**Methods:**

we will use the Cochrane Handbook for Systematic Reviews of Interventions to guide whole this review process. We will consider parallel or cluster-randomised controlled trials (RCTs), quasi-RCTs, and cohort studies. We will search CENTRAL, MEDLINE, Embase, PsycINFO, LILACS and IBECS. We will also conduct a search of ClinicalTrials.gov and the ICTRP for ongoing or unpublished trials. Each research step will involve at least two independent reviewers. We will create a table, using GRADE pro GDT software, reporting the pooled effect estimates for the following outcomes: all-cause mortality, lower limb amputation, adverse events, walking distance, clinical severity, vessel or graft secondary patency, and QoL.

**Conclusions:**

we will assess these outcomes according to the five GRADE considerations to assess the certainty of the body of evidence for these outcomes, and to draw conclusions about the certainty of the evidence within the review.

## RESUMO

**Introdução:**

a doença arterial periférica tem o tabagismo como principal fator de risco vascular evitável. Entretanto, a maioria dos estudos não destaca o tabagismo como principal variável de exposição.

**Objetivos:**

avaliar o impacto das intervenções de cessação do tabagismo versus comparador ativo, placebo ou nenhuma intervenção, nos desfechos da doença arterial periférica.

**Métodos:**

usaremos o Cochrane Handbook for Systematic Review of Interventions para orientar todo este processo de revisão. Consideraremos ensaios controlados paralelos ou randomizados por cluster (ECRs), quase-ECRs e estudos de coorte. Buscaremos no CENTRAL, MEDLINE, Embase, PsycINFO, LILACS e IBECS. ClinicalTrials.gov e ICTRP serão consultados para ensaios em andamento ou não publicados. Criaremos uma tabela, usando o software GRADE pro GDT, relatando as estimativas de efeito agrupado para os seguintes desfechos: mortalidade por todas as causas, amputação de membro inferior, eventos adversos, distância percorrida, gravidade clínica, permeabilidade secundária do vaso ou enxerto e qualidade de vida. Avaliaremos esses resultados de acordo com as cinco considerações GRADE para avaliar a certeza do corpo de evidências para esses resultados e tirar conclusões sobre a certeza das evidências na revisão.

## INTRODUCTION

According to the World Health Organization (WHO), there are 1.3 billion smokers in the world^1^. Peripheral artery disease (PAD) has smoking as its main preventable vascular risk factor^2^. Worldwide, there are more than 200 million people with PAD and its prevalence has increased by 23.5% in ten years^3^.

PAD can be an asymptomatic disease or present with typical symptoms, such as intermittent claudication or pain in the legs caused by walking. This disease can be conceptualized as a total or partial occlusion of one or more peripheral arteries^4^.

Measurable disease, whether asymptomatic or with atypical symptoms, is present in more than 8 million Americans, a worrisome fact, since coronary and cerebrovascular diseases are associated with PAD (symptomatic or asymptomatic), with a very high mortality risk^5^ ^-^ ^7^. One study applied multiple regression to risk factors on PAD measures and showed that the only consistent difference was that smoking increased the risk of PAD (odds ratio [OR] range 1.8-5.6) more than of heart disease (OR range 1.1-1.6)^3^ ^,^ ^4^.

Most studies do not focus on smoking as the main exposure variable^8^. Furthermore, there is great variation in the strength of the relationship among studies, with relative risks for current smoking ranging from 1.6 to 10.2^9^. Therefore, despite the large number of studies, more detailed evaluations of smoking as a risk factor for PAD are limited. 

Smoking cessation has shown correlation with a reduced risk of developing PAD. However, it is estimated that this reduction takes more than 20 years to reach the risk level of people who have never smoked^10^. Young adult smokers may already be increasing their risk of PAD many years before its clinical presentation, as smoking increases the risk of asymptomatic PAD^3^.

The high prevalence of atherosclerosis in smokers is related to several pathophysiological mechanisms, such as changes in lipoprotein metabolism, endothelial dysfunction, and in coagulation and platelet function. The risk of cardiovascular morbidity and mortality is reduced with smoking cessation, in addition to improving the functional capacity of patients with PAD^11^. For this, medical advice and nicotine replacement therapy are useful and strongly recommended^12^.

A cohort of 5,306 black participants who were current smokers had, after adjustment for covariates, an increased risk of an ankle-brachial index <1 (OR 2.2, 95% CI 1.5-3.3) and an increased risk of involvement of the abdominal aorta (OR 8.4, 95% CI 5.8-12.0) and aortoiliac segment (OR 9.6, 95% CI 6.7-13.7). Those who smoked more than 20 cigarettes a day were more likely to have asymptomatic or mildly symptomatic PAD compared with those who smoked less, suggesting a dose-dependent relationship^13^.

Current smokers or former smokers accounted for more than 80% of patients with PAD^14^ ^,^ ^15^. Current smokers with PAD have more than twice the cardiovascular mortality rate compared with patients with PAD who have never smoked^16^.

The Atherosclerosis Risk in Communities study analyzed 13,355 participants aged 45 to 64 years without PAD, stroke, or coronary artery disease (CAD) at baseline (1987 to 1989). Cox models were used to analyze the incidence of PAD with associations of smoking parameters (pack-year and cessation) and compared with the incidence of CAD and stroke. During 26 years of follow-up, there were 1,798 cases of CAD, 1,106 strokes, and 492 cases of PAD. In this study, a relationship was found between the amount of smoking and the years of smoking, mainly for PAD. With longer smoking cessation, a lower risk of CAD, stroke, and PAD was identified, up to 30 years for PAD and 20 years for CAD. Smoking prevention and early cessation are emphasized by the study, which also indicates that public statements that take PAD into account are necessary to recognize the impact of smoking on general cardiovascular health^17^.

Therefore, we intend to highlight smoking as the main exposure variable. We will evaluate, through a systematic literature review, the impact of smoking cessation interventions versus active comparator, placebo or no intervention, on peripheral arterial disease outcomes.

## OBJECTIVES

To assess the impact of smoking cessation interventions versus active comparator, placebo or no intervention, on peripheral arterial disease outcomes.

## METHODS

### Eligibility criteria

#### Types of studies

We used the Cochrane Handbook for Systematic Review of Interventions to guide this entire review process^18^. We will consider parallel or cluster-randomized controlled trials (RCTs), quasi-RCTs, and cohort studies. We will not consider studies without a comparison group. In future updates of this review, when at least 400 participants are included from RCTs, we will no longer consider non-randomized studies (NRCTs) for inclusion. We will consider all other types of studies irrelevant for this review.

In case of insufficient evidence available from RCTs, we will include NRCTs from interventions, including quasi-randomized controlled trials. In case of insufficient evidence available from prospective RCTs, quasi-RCTs, or NRCTs, we will include retrospective observational studies with a control group.

Any prospective comparative cohort of clinically diagnosed cases of PAD will be included if the cohort includes current smokers at baseline. Smoking status should be measured on at least two occasions to see which smokers have quit. The ‘control group’ will therefore consist of those who continued to smoke, to be compared with those who quit. Only studies with a minimum duration of six months will be considered.

#### Types of participants

We will include male and female participants of all ages, both hospitalized and non-hospitalized, with a confirmed diagnosis of PAD at any stage. Ideally, these will be classified at least with the objective test ABI (ankle-brachial index), which is the ratio of systolic blood pressure at the ankle to that of the upper arm. An ABI ≤0.90 is commonly used to diagnose PAD^4^. We will also consider a diagnosis of PAD when confirmed by a point-of-care clinical test that demonstrates one or more of the following signs:

- Ankle systolic pressure (ASP) <80mmHg;
- Toe systolic pressure <40mmHg;
- Transcutaneous oxygen tension (TcO2) <40mmHg.

Other objective exams for the diagnosis of PAD can be used, such as duplex ultrasonography or angiography (by computed tomography, magnetic resonance, or digital subtraction)^19^ ^,^ ^20^. Furthermore, any assertion by the author that the patients were ‘smokers’ and that they ‘stopped smoking’ will be accepted.

#### Types of interventions

The intervention will be any smoking cessation. We will include any definition of smoking cessation by the included studies. We will prefer more rigorous measures and intention-to-treat rather than a full case analysis where multiple definitions of cessation are reported.

### Information sources

#### Search methods for identifying studies

We will search CENTRAL, MEDLINE, Embase, PsycINFO, LILACS, and IBECS. We will adapt the MEDLINE preliminary search strategy for use in other databases (Table 4). We will not apply any RCT filters to any databases, but we will select the study design manually because we will also consider NRCT for inclusion in this review. We will also perform a search of ClinicalTrials.gov and the International Clinical Trials Registry Platform (ICTRP) through the WHO portal for ongoing or unpublished trials.

We will search all databases from inception to the present and will not restrict publication language or publication status. If necessary, we will request assistance from native speakers of specific languages, through the Cochrane Task Exchange (taskexchange.cochrane.org), for data extraction and translation of the original manuscripts. We will only consider the adverse effects described in the included studies.

#### Searching other resources

We will:

- check bibliographies of included studies and any relevant systematic reviews identified for further references to studies and search Google Scholar;
- when necessary, contact the original authors of the study;
- when necessary, contact experts in the field for additional information on relevant studies, using a standard letter template; and
- conduct a search of various gray literature sources, databases of dissertations, theses, and conference abstracts.

#### Selection of studies

Two reviewers will independently review the titles and abstracts of all potential studies we identify as a result of the search and code them as ‘retrieve’ (eligible or potentially eligible/unclear) or ‘do not retrieve’ using the Covidence tool^21^. We will retrieve the full text study reports/publications, and two reviewers will independently review the full text and identify the studies for inclusion, as well as identifying and recording the reasons for excluding the ineligible studies. We will identify and exclude duplicates and group multiple reports from the same study so that each study, rather than each report, is the unit of interest in the review. We will record the selection process in sufficient detail to fill in a PRISMA flow diagram and the ‘Characteristics of Excluded Studies’ table^22^. We will consider studies reported as full text, those published only as abstracts, and unpublished data. We will consider conference abstracts and proceedings if they are eligible and have usable data. We will consider abstracts and full texts in all languages for inclusion.

We will resolve any disagreements during any phase of this study through discussion or, if necessary, request arbitration from a third review author.

### Data extraction and management

Two reviewers will test the data extraction form and make appropriate changes. Two reviewers will extract data from each study independently.

We will gather the following data from each study:

- Study design;
- Analysis method;
- Outcome measures;
- Length of follow-up;
- Sample size at baseline and follow-up;
- Type of population;
- Percentage (%) gender;
- Mean age (standard deviation (SD));
- Adjusted covariates;
- Motivation to quit;
- Intervention(s) used (if relevant);
- Risk of bias with ROBINS-I^23^ ;
- Data to calculate standardized mean difference (SMD) in PAD outcomes: for each group - mean at baseline and follow-up, mean change from baseline to follow-up, and difference in mean change from baseline to follow-up and variance;
- Data to calculate the risk of PAD outcomes: for each group - number of participants in the baseline control group, number of participants in the baseline exposure group, number of participants with PAD in the follow-up exposure group, and number of participants with PAD in the control group at follow-up; and
- Study funding sources and authors’ declarations of interest.

## RESULTS AND PRIORITIZATION

### Primary results

- All-cause mortality: assessing mortality as a dichotomous variable. We will not consider time to all-cause mortality.
- Lower limb amputation: proportion of people who had a lower limb amputation, at any level, during the follow-up period.
- Adverse Events: We will consider all adverse events separately as individual outcomes.

### Secondary outcomes

- Walking distance, in meters, on a treadmill or by another walking test;
- Clinical severity scales: any validated clinical scales, such as Fontaine (Table 1), Rutherford (Table 2) or the WIFI classification (Table 3)^24^ will be considered^25^;
- Vessel or graft secondary patency: the patency after an intervention performed to treat a graft vessel after thrombosis; and
- Quality of life (QoL): measured by any validated instrument, such as the Short Form 36 (SF - 36) or the EuroQol^26^ questionnaires.

**Table 1:** Fontaine classification for peripheral artery disease (Fontaine 1954).

Stage	Description
I	Asymptomatic
II	Mild claudication
IIa	Claudication distance >200 meters
IIb	Claudication distance <200 meters
III	Pain at rest (especially at night)
IV	Ulceration and/or gangrene of the limb

**Table 2:** Rutherford classification for peripheral artery disease.

Grade	Category	Description	Objective criteria
0	0	Asymptomatic - without hemodynamically significant occlusive disease	Normal treadmill or reactive hyperemia test
	1	Mild claudication	Complete the exercise on a treadmill; AP after exercise >50mmHg but at least 20mmHg below resting value
I	2	Moderate claudication	Between categories 1 and 3
	3	Severe claudication	Does not complete standard treadmill exercise and AP after exercise <50mmHg
II	4	Ischemic pain at rest	AP at rest <40mmHg, ankle or metatarsal PVR flat or slightly pulsating; TP <30mmHg
III	5	Minor tissue loss - non-healing ulcer, focal gangrene with diffuse foot ischemia	AP at rest <60mmHg, ankle or metatarsal PVR flat or slightly pulsating; TP <40mmHg
	6	Major tissue loss - extending above TM level, functional foot no longer recoverable	Same as category 5

AP: ankle pressure. PVR: pulse volume recording. TM: transmetatarsal. TP: toe pressure.

**Table 3:** Society for Vascular Surgery WIFI classification (wound, ischemia and foot infection).

Wound			
Grade	Ulcer	Gangrene	
0	No ulcer	No gangrene	
1	Small, shallow ulcer on the distal leg or foot; no exposed bone, except limited to the distal phalanx	No gangrene	
2	Deeper ulcer with exposed bone, joint, or tendon usually not involving the heel; shallow heel ulcer without calcaneal involvement	Gangrenous changes limited to digits	
3	Extensive and deep ulcer involving forefoot and/or midfoot; full thickness deep heel ulcer ± calcaneal involvement	Extensive gangrene involving the forefoot/midfoot; full-thickness heel necrosis ± calcaneal involvement	
Ischemia			
Grade	ABI	Ankle systolic blood pressure	TP, TcPO2
0	≥0,80 0,6 - 0,79 0,4 - 0,59 ≤0,39	>100mmHg	≥60mmHg
1		70 - 100mmHg	40 - 59mmHg
2		50 - 70mmHg	30 - 39mmHg
3		<50mmHg	<30mmHg
Infection			
Grade	Clinical manifestation of infection		
0	No symptoms or signs of infection Infection present, defined by the presence of at least two of the following: • Local swelling or hardening • Erythema 0.5-2cm around the ulcer • Local tenderness or pain • Local heat • Purulent discharge (thick, opaque to white, or bloody discharge)		
1	Local infection involving only the skin and subcutaneous tissue Exclude other causes of inflammatory skin response (trauma, gout, acute Charcot’s syndrome, fracture, thrombosis, and venous stasis)		
2	Local infection with erythema >2 cm or involving structures deeper than the skin and subcutaneous tissues, without signs of systemic inflammatory response		
3	No signs of systemic inflammatory response Local infection with signs of SIRS, manifested by two or more of the following: • Temperature >38 or <36°C	• Heart rate >90 beats/min • Respiratory rate >20 breaths/min or PaCO2 <32mmHg	• White blood cell count >12,000 or <4,000 cu/mm or 10% immature bands

ABI: ankle-brachial index. PaCO2: arterial carbon dioxide partial pressure. SIRS: systemic inflammatory response syndrome. TP: toe pressure. TcPO2 : transcutaneous oxygen pressure.

**Table 4:** Search strategy draft (MEDLINE via PubMed).

#	Query	Results
#1	"Tobacco Use Cessation Products"[Mesh] OR (Nicotine Polacril *) OR (Nicotine Replacement Product*) OR (Nicotine Nasal Spray*) OR (Nicotine Inhalant*) OR (Nicotine Lozenge*) OR (Nicotine Patch) OR (Nicotine Transdermal Patch) OR (Smoking Cessation Product*)	4.936
#2	"Tobacco Use Cessation"[Mesh] OR (Cessation* Tobacco Use) OR (Tobacco Cessation) OR (Smokeless Tobacco Cessation)	17.472
#3	"Smoking Cessation"[Mesh] OR (Cessation* Smoking) OR (Stopping Smoking) OR (Giving Up Smoking*) OR (Quitting Smoking)	38.527
#4	"Peripheral Arterial Disease"[Mesh] OR (Arter* Disease* Peripheral)	28.826
#5	"Arterial Occlusive Diseases"[Mesh] OR (Arterial Occlusive Disease*) OR (Arterial Obstructive Disease*)	214.057
#6	= #1 OR #2 OR #3 “Tobacco Use Cessation Products”[Mesh] OR (Nicotine Polacril*) OR (Nicotine Replacement Product*) OR (Nicotine Nasal Spray*) OR (Nicotine Inhalant*) OR (Nicotine Lozenge*) OR (Nicotine Patch) OR (Nicotine Transdermal Patch) OR (Smoking Cessation Product*) OR “Tobacco Use Cessation”[Mesh] OR (Cessation* Tobacco Use) OR (Tobacco Cessation) OR (Smokeless Tobacco Cessation) OR “Smoking Cessation”[Mesh] OR (Cessation* Smoking) OR (Stopping Smoking) OR (Giving Up Smoking*) OR (Quitting Smoking)	40.611
#7	= #4 OR #5 “Peripheral Arterial Disease”[Mesh] OR (Arter* Disease* Peripheral) OR “Arterial Occlusive Diseases”[Mesh] OR (Arterial Occlusive Disease*) OR (Arterial Obstructive Disease*)	228.320
#8	= #6 AND #7 (“Tobacco Use Cessation Products”[Mesh] OR (Nicotine Polacril *) OR (Nicotine Replacement Product*) OR (Nicotine Nasal Spray*) OR (Nicotine Inhalant*) OR (Nicotine Lozenge*) OR (Nicotine Patch) OR (Nicotine Transdermal Patch) OR (Smoking Cessation Product*) OR “Tobacco Use Cessation”[Mesh] OR (Cessation* Tobacco Use) OR (Tobacco Cessation) OR (Smokeless Tobacco Cessation) OR “Smoking Cessation”[Mesh] OR (Cessation* Smoking ) OR (Stopping Smoking) OR (Giving Up Smoking*) OR (Quitting Smoking)) AND (“Peripheral Arterial Disease”[Mesh] OR ( Arter * Disease* Peripheral) OR “Arterial Occlusive Diseases”[Mesh] OR (Arterial Occlusive Disease*) OR (Arterial Obstructive Disease*))	841
#9	= #8 AND CLINICAL QUERIES	rct = 593 SR = 87

### Assessment of the risk of bias in the included studies

For data from RCTs, we will use the ‘Risk of Bias’ 1.0 (RoB 1)^27^ tool. For data from prospective or retrospective NRCTs, we will use the ROBINS-I^23^ tool. If we include only RCTs and quasi-RCTs, we will assess the risk of bias for quasi-RCTs with the RoB 1 tool. We will consider the following confounders for the ROBINS-I domain assessment under ‘confounding’ and use the Robvis tool to create the ‘risk of bias’ graphs for NRCTs^28^:

- participants who already use antithrombotic drugs; and
- participants undergoing surgery during the follow-up period.

### Randomized controlled trials

Two Review authors will assess the risk of bias for each study using the criteria described in the Cochrane Handbook for Systematic Reviews of Interventions^27^ for RCTs (RoB1 tool), according to the following domains:

- Random sequence generation;
- Allocation concealment;
- Blinding of participants and personnel;
- Blinding of outcome assessment;
- Incomplete outcome data;
- Selective outcome reporting; and
- Other biases.

In cluster-randomized studies, we will consider particular biases as recommended by section 8.15.1.1 of the Cochrane Handbook for Systematic Reviews of Interventions: recruitment bias, baseline imbalance, loss of clusters, incorrect analysis, and comparability with individually randomized trials^27^. We will score each potential source of bias as high, low, or unclear, and provide a citation from the study report along with a rationale for our judgment in the ‘Risk of Bias’ table. We will summarize the ‘Risk of Bias’ judgments across different studies for each of the domains listed.

When considering treatment effects, we will take into account the risk of bias for the studies that contributed to this result.

We will base the overall judgment of bias from the included RCTs on the three domains of the RoB1 tool: generation of adequate sequence, blinding of outcome raters, and selective reporting of results. A low-risk RCT in all of these domains will be labeled a low-risk study. A high-risk RCT in one of these domains will be labeled a high-risk study. If there is no clear information about the risk of bias for one or more key domains but the RCT is not high-risk for any domain, we will indicate that the risk of bias in the study is unclear.

### Non-randomized studies

Using the ROBINS-I tool, we will assess the risk of bias of quasi-RCTs and NRCTs based on the following domains^23^:

- Confounding;
- Selection of participants into the study;
- Classification of interventions;
- Deviations from the intended intervention;
- Missing data;
- Measurement of outcomes; and
- Selection of the reported result.

We will use our ‘Risk of Bias’ judgments for quasi-RCTs and NRCTs to label the outcomes for each comparison in these domains as ‘critical risk’, ‘severe risk’, ‘moderate risk’, ‘low risk’, or ‘no information’. We will judge the overall risk of bias (across domains) as the worst judgment across all domains.

### Measures of treatment effect

#### Dichotomous data

We will calculate the risk ratio (RR) and 95% confidence intervals (CI).

#### Continuous data

We will calculate mean differences (MD) and 95% CIs between treatment groups where studies report the same results. Where similar results are reported on different scales, we will calculate the SMD and its 95% CI. To interpret the SMD, we will use the following thresholds:

- SMD <0.2 = trivial or no effect;
- SMD ≥0.2 and <0.5 = small effect;
- SMD ≥0.5 and <0.8 = moderate effect;
- SMD ≥0.8 = large effect.

### Unit of Analysis Problems

The planned unit of analysis is the individual smoker, unless the study is cluster-randomized, in which case the relevant cluster (eg, community, institution, or caregiver) is the unit of analysis. In the case of a cluster-randomized study using the individual as the unit of analysis, we will report the trialists’ methods for adjusting the analyzes for intraclass correlation. For cluster-RCTs, we will present cluster-adjusted results, extract the 95% CI, and use the inverse variance method to combine trials.

### Data summary

We will pool the changing SMDs and measures of variance from individual studies using a generic inverse variance random effects model. An SMD greater than zero will indicate that quitting smoking is associated with worse PAD at follow-up.

We will pool risk ratios (RRs) and measures of variance calculated for individual studies using a Mantel-Haenszel random effects meta-analysis. An RR greater than one will indicate that people who stop smoking have a higher risk of PAD at follow-up.

We will carry out meta-analyses of SMD and RR for each outcome separately using the Revman 2020^29^.

### Evaluation of heterogeneity

We will quantify statistical heterogeneity using I², which describes the percentage (%) of inter-study variability due to heterogeneity rather than chance. We will consider the I² value between 50% and 90% as substantial heterogeneity, and should it be above 90%, we will assess whether it is appropriate to report a pooled analysis^30^.

### Subgroup analysis and investigation of heterogeneity

We will conduct subgroup analyses, where appropriate, to explore the impact of different variables on review results. This helps to identify and investigate unexplained sources of heterogeneity.

### Sensitivity analysis

We will perform sensitivity analyzes to test the robustness of estimates across different eligibility criteria, such as participants, interventions, comparators, outcome characteristics, and methodology in study designs. We also plan to use sensitivity analysis to examine the impact of data characteristics, such as measurement level (continuous or ordinal), time to event, pooled or cross-run correlation coefficients, and methods of analysis.

### Dealing with lost data

We will contact study investigators for missing numerical data where possible. We shall use the RevMan 5 calculator to try to calculate missing standard deviations using other test data. Where this is not possible and missing data are considered to be seriously biased, we will explore the impact of including such studies on the overall assessment of results by a sensitivity analysis. For all outcomes, we will follow intention-to-treat (ITT) principles to the highest possible degree, i.e., we will analyze participants in their randomized group, regardless of which intervention they received. We will use available case data for the denominator if ITT data is not available. We will estimate the DM using the method reported by Wan et al.^31^ to convert median and interquartile range (IQR) into MD and CI. When this is not possible, we will narratively describe the skewed data reported as medians and IQRs.

### Assessment of reporting biases

We will examine funnel plots for evidence of asymmetry and perform Egger’s tests for evidence of small study bias where there are 10 or more studies contributing to a comparison.

### Table ‘Summary of findings’ and GRADE

We will create a ‘Summary of Findings’ table, using the GRADE pro GDT software, reporting the pooled effect estimates for the following outcomes: all-cause mortality, lower limb amputation, adverse events, distance traveled, clinical severity, secondary patency of the vessel or graft, and quality of life. We will evaluate these results according to the five GRADE considerations to assess the certainty of the body of evidence for these results and draw conclusions about the certainty of the evidence in the review text^32^.

## CONCLUSIONS

We will base our conclusions only on the findings of the quantitative or narrative summary of the studies included in this review. Furthermore, we will avoid making any recommendations for clinical practice, and our implications will suggest priorities for future research and outline the remaining uncertainties in the area.
